# Impact of Coronavirus Outbreaks on Science and Society: Insights from Temporal Bibliometry of SARS and COVID-19

**DOI:** 10.3390/e23050626

**Published:** 2021-05-18

**Authors:** Ramya Gupta, Abhishek Prasad, Suresh Babu, Gitanjali Yadav

**Affiliations:** 1 National Institute of Plant Genome Research, New Delhi 110067, India; ramyagupta96@gmail.com (R.G.); abhishek1992@nipgr.ac.in (A.P.); 2 School of Human Ecology, Ambedkar University Delhi, Delhi 110007, India; suresh@aud.ac.in; 3 Department of Plant Sciences, University of Cambridge, Cambridge CB23EA, UK

**Keywords:** bibliometry, coronavirus, text and data mining, SARS, MERS, COVID-19

## Abstract

A global event such as the COVID-19 crisis presents new, often unexpected responses that are fascinating to investigate from both scientific and social standpoints. Despite several documented similarities, the coronavirus pandemic is clearly distinct from the 1918 flu pandemic in terms of our exponentially increased, almost instantaneous ability to access/share information, offering an unprecedented opportunity to visualise rippling effects of global events across space and time. Personal devices provide “big data” on people’s movement, the environment and economic trends, while access to the unprecedented flurry in scientific publications and media posts provides a measure of the response of the educated world to the crisis. Most bibliometric (co-authorship, co-citation, or bibliographic coupling) analyses ignore the time dimension, but COVID-19 has made it possible to perform a detailed temporal investigation into the pandemic. Here, we report a comprehensive network analysis based on more than 20,000 published documents on viral epidemics, authored by over 75,000 individuals from 140 nations in the past one year of the crisis. Unlike the 1918 flu pandemic, access to published data over the past two decades enabled a comparison of publishing trends between the ongoing COVID-19 pandemic and those of the 2003 SARS epidemic to study changes in thematic foci and societal pressures dictating research over the course of a crisis.

## 1. Introduction

Unlike the 1918 flu pandemic, COVID-19 has revealed how the Internet of Things (IOT) can impact the ability of a society to cope and survive. A number of significant technology changes have come together to enable this, as more machines and people are being equipped with networked sensors to report their status, receive instructions, and even take action based on the information they receive [1]. Data are constantly being collected and documented from an estimated 10 billion mobile phones, over 2000 satellites and more than 25 billion digital sensors to monitor and quantify shifts in social and economic activities in response to the pandemic [2]. Such “big data” is helping steer scientific research towards addressing the crisis and return to normalcy, and strongly impacts the state’s inherent capacity to make informed policy decisions based on social trends and scientific evidence [3,4,5]. The scientific world has witnessed an unprecedented flurry in publications ever since the first report of a new coronavirus disease outbreak at the end of December 2019, which was followed by its rapid global spread, leading up to the announcement by the World Health Organization on 11 March 2020 that COVID-19 had become a global pandemic [6,7,8]. These publications are often from the most infected countries such as the United States, China and Italy, but the evolution of research topics is in real time and can be assessed as such to understand how the world is addressing the crisis. Earlier in this millennium, the world witnessed two other epidemics caused by members of the coronavirus group, namely, the severe acute respiratory syndrome coronavirus (SARS-CoV) and Middle East respiratory syndrome coronavirus (MERS-CoV) [9,10,11]. After each outbreak, multi-disciplinary research into diverse aspects of the diseases was undertaken, including virology, immunology, transmission, diagnosis, management and vaccine development. The extraction and analysis of knowledge from this scholarly corpus can add valuable insights and enable the synthesis of existing research findings while delineating new directions for future research [12]. Our first objective in this work was to search the COVID-19-related scholarly corpus, and compare it with the previous epidemic, in order to establish the value of “Big Data” in the present crisis that was not observed during the earlier epidemics (and was near impossible in the 1918 flu pandemic). Our second objective was to identify global trends in COVID-19 research, including the distribution of inter-institutional and country collaborations, and most importantly, research hotspots. Both of these objectives have been achieved by way of rigorous bibliometry analyses.

Rigorous bibliometric methods can identify coherent clusters in existing research that can serve as reference points and identify knowledge gaps that remain to be addressed [13]. In this regard, the visualisation and conceptualisation of a complex co-citation corpus as networks enables the derivation of biologically significant inferences from systematic analysis of detailed conceptual relationships [14]. Very recently, we developed a new decision support system based on recursive partitioning of bibliometric evidence to simplify exploratory literature reviews, enabling the rational design of research objectives for scholars, as well as the development of comprehensive grant proposals that address gaps in research [15]. In this work, we use this method, taking into account the time dimension (on a quarterly basis), to gain a near-real time glimpse into how the pandemic is impacting scientific research in different ways across spatial scales. 

The basic parameters used to plot bibliometric networks include the number of documents, number of sources (journals, books, etc.) in which the documents have been published, number of Keywords Plus, number of authors, publication period, and the collaboration index. Keywords Plus by Clarivate Analytics’ Web of Science includes recurring phrases from all the titles in a document’s reference list [16]. The collaboration index is calculated as the number of authors of multi-author documents divided by the number of multi-author documents. It provides a quantitative metric to measure research collaboration [17]. 

A useful tool to analyse the contribution of sources in a collection is Bradford’s law. The law categorises the sources contributing to the research in a particular field into “zones”. The top sources in the list are categorised as “core sources” or “Zone 1” sources that are most frequently cited in that field. Zone 2 and Zone 3 contain less frequently cited sources. Then, the number of sources in each zone can be calculated as 1, n, n^2^, etc. [18]. Another such law is Lotka’s law. It is used to measure author productivity and contribution to the research in a field. It is a modified inverse square law that can be used to calculate how many authors will publish any fixed number of documents in a field [19]. The diversity of research themes within a subject area can be analysed using co-occurrence networks plotted for Keywords, or collaboration networks for countries and institutes that often reveal trends in research collaboration. Another parameter used to quantify international collaboration (in addition to the collaboration index) is the Multiple Country Publication Ratio (MCP Ratio). The MCP is identified as a publication where at least one author is from a country different from that of the other authors. The MCP Ratio is then calculated as the number of MCPs for a country divided by the total number of publications the country has contributed to the collection [17]. 

In summary, this work develops a conceptual framework integrating the three dimensions of time, space and scientific evidence to enable a reassessment of the nature, dynamics and nuances of bibliometric networks based on published data. We find interesting distinctions between the first 100 days of the two outbreaks SARS and COVID-19, in terms of international cooperation as well as keyword trend-shifts that suggest a much greater extent of utilisation of big data in 2020 as compared to 2003. We also observe a quarterly emergence/change in keywords or “research hotspots” during the first year of the pandemic, revealing how the focus of the world is shifting from disease biology to patient wellness. Unfortunately, we also find that the extraordinary amount of data available today has little impact on the policy process at local or global scales. These insights also bring forth an urgent need for cooperation between governments and scientific researchers globally to jointly fight the epidemic, and harness the data revolution more responsibly and carefully, in order to achieve a new normal that can be more resilient, safer and sustainable.

## 2. Materials and Methods

Bibliometry is an academic science founded on a set of statistical methods, which can be used to analyse scientific big data quantitatively and their evolution over time for the discovery of the underlying structure of the data. Network structure is often used to model the interaction among authors, papers/documents/articles, references, keywords, etc. All data in this work were analysed using the R Bibliometrix and ggplot2 packages [17,20], an open-source software for automating the stages of data-analysis and data-visualisation. Data were collected using the Web of Science Core Collection search tool. The search terms used were: “SARS”, “coronavirus”, “SARS-CoV-2” and “COVID-19”. All data from the year 2001 onwards were downloaded. This was performed on 17 April 2020. The data were organised into three groups:

1. Group A: 100 days from 1 January 2020; keywords “SARS-CoV-2” OR “COVID-19”;
2. Group B: From 2001–2020 for search terms “SARS” OR “coronavirus”;
3. Group C: 100 days from 1 January 2003; keywords “SARS” OR “coronavirus”.

Group C was used to compare the publication trends of the first 100 days of the coronavirus pandemic to those of the 2003 SARS epidemic, and care was taken not to use the term “coronavirus” for Group A in order to avoid any SARS-related work in 2020. Another round of data collection was undertaken in January 2021. This time, the search term used was “COVID-19” alone, since “SARS-CoV-2” keyword matches were found to overlap with those of “COVID-19”. All data from 1 January 2020 to 31 December 2020 were downloaded and organised into four temporal sections as follows:

1. Q1: all data from January to March 2020;
2. Q2: all data from April to June 2020;
3. Q3: all data from July to September 2020;
4. Q4: all data from October to December 2020.

The data from the four quarters were used to compare publishing trends over the course of the year of the pandemic. 

### 2.1. Descriptive Analyses 

Bibliometry is a very useful tool for displaying and analysing the intellectual, conceptual and social structures of research as well as their evolution and dynamical aspects. Descriptive bibliometric methods help to map the science and are very useful for systematic research synthesis. Descriptive analysis provides the main features of the collections being investigated; in our case Group A, B and C as well as the quarterly collections for 2020 (Q1–Q4). For each collection, it returns snapshots about the annual research development, the top “k” productive authors, papers, countries and most relevant keywords.

### 2.2. Conceptual Structure Analyses: Co-Citation and Co-Word Networks

Citation analysis is one of the main classic techniques in bibliometry. It shows the structure of a specific field through the linkages between nodes (e.g., authors, papers, journal), while the edges can be differently interpretated depending on the network type, which are namely co-citation, direct citation, bibliographic coupling. The useful dimensions to comment the co-citation networks are: (i) centrality and peripherality of nodes, (ii) their proximity and distance, (iii) strength of ties, (iv) clusters, (v) bridging contributions. This also includes the generation of historiographs, built on direct citations, drawing intellectual linkages in a historical order. Cited works of thousands of authors contained in a collection of published scientific articles are sufficient for reconstructing the historiographic structure of the field, calling out the basic works in it. Co-word networks show the conceptual structure that uncovers links between concepts through keyword term co-occurrences. Conceptual structure is often used to understand the topics covered by scholars and identify what are the most important and the most recent issues. Dividing the whole timespan into different time slices and comparing the conceptual structures is useful to analyse the evolution of topics over time. This is not merely limited to keywords, but also the terms in the articles’ titles and abstracts. This is performed through network analysis or correspondence analysis (CA) or multiple correspondence analysis (MCA). CA and MCA visualise the conceptual structure in a two-dimensional plot.

## 3. Results

### 3.1. Annual Scientific Production Shows Peaks for Past Epidemics

**Figure 1:**
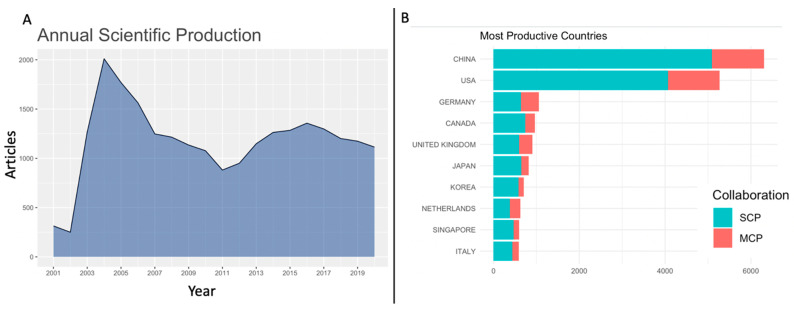
Summary of Group B (20 years) data, including (**A**) annual scientific production curve and (**B**) topmost productive countries showing Single Country Publications (blue bars) and Multiple Country Publications (orange bars).

Figure 1 shows the annual scientific production curve for the past 20 years of published literature (Group B data), and clear peaks are visible during the Severe Acute Respiratory Syndrome (SARS-CoV) epidemic (2003–2004) and the beginning of the Middle East Respiratory Syndrome (MERS-CoV) epidemic (2012–2013). Furthermore, China and the USA are among the most productive countries, as reflected by their numbers of cases of infection, especially for the SARS-CoV epidemic that affected the Far East and North America almost exclusively [21]. This trend helped us understand the most productive countries assessed for Group A and correlate these with cases of reported infections in later sections of this work. The ASP curve also served to identify a starting point to compare publishing trends during the ongoing COVID-19 pandemic with those of past coronavirus-related epidemics, especially 2003 SARs-CoV since it showed the highest ASP in the entire 20 year corpus. The subsequent sections compare patterns observed in the first 100 days of the 2003 SARS epidemic and 2020 COVID-19 pandemic. Data were collected as described in Materials and Methods and the two groups are henceforth referred to as Group A (for 2020) and Group C (for 2003).

### 3.2. Greater Volume of Work in COVID-19 Pandemic as Compared to SARS

**Figure 2:**
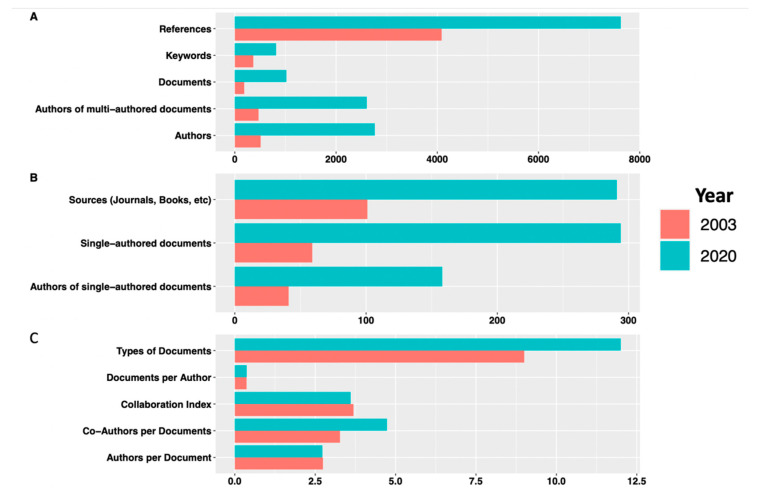
Comparison of the first 100 days of bibliography for Group C SARS 2003 (orange bars) and Group A COVID-19 (blue bars). The bar plots represent Table 1 data variables in three sections with (**A**) general information, (**B**) source and author data and (**C**) documents and collaborations.

The complete bibliometric data and information collected for the Groups A, B and C are depicted in Table 1, whereas Figure 2 provides a more visible comparison between the 2003 and 2020 datasets. As can be seen in Figure 2, it was observed that the number of documents published during the first 100 days of the coronavirus pandemic (Group A) was 5.4 times the number of documents published in the first 100 days of the SARS epidemic (Group C), a significant rise, even after normalising for background noise of the previous years, respectively (using Group B data, which showed 50 papers in 2002; 200 in 2019). This is despite the two datasets having the same baseline documents per author and collaboration index for authors, a metric considered better than traditional metrices such as H-index as they are able to account for collaboration, which can have a strong bearing on the estimated individual scientific impact [22]. Figure 2 also reveals that the number of documents published immediately after the COVID-19 outbreak was almost ten times the corresponding number for the SARS epidemic, while the number of authors publishing their work in the first 100 days of the COVID-19 pandemic was about six times higher than for the corresponding period during SARS Group C, suggesting that during COVID-19, (a) significantly more authors contributed to the surge of publications, and (b) a higher number of journals contributed to the collections, as compared to SARS-CoV.

Interestingly, Table 1 and Figure 2 reveal a consistently higher share of the 2020 scientific corpus as compared to the 2003 coronavirus corpus. This is reflected by the much higher share of the 2020 corpus in terms of references cited (almost double in Group A COVID-19), authors of documents both single-authored (4× increase) and multi-authored (6× increase), as well as five times more single authored documents in Group A. The data also show much wider thematic focus of the 2020 publications, as evident from a greater number of keywords in Group A COVID-19 data (almost three times as compared to Group C SARS data), and in retrospect, this pattern appears to be specific to the global crisis of 2020 with a worldwide surge in research aligning with diverse aspects of the pandemic. A country-wise comparison of the number of documents contributed by each group revealed one overall trend: more countries were involved in publishing at the start of the coronavirus pandemic than at the start of the SARS epidemic. Several African, Eastern European, and South American countries started publishing early on during the coronavirus pandemic. This was not seen during the SARS epidemic, and may be a reflection of the limited geographical impact of the SARS epidemic as compared to COVID-19, the former being almost exclusively an affliction in the Far East and North America [21]. At any cost, these trends merit a detailed investigation of the 2020 publication patterns, as has been attempted in the subsequent sections in terms of quarterly bibliography data over the course of 2020, grouped into Quarters Q1–Q4 as described in Materials and Methods. Within these four datasets, we noted the highest collaboration index during Q1, when the pandemic was still very new across the globe, suggesting that at the beginning of the pandemic, a large number of authors came together to collaborate in order to address the crisis, but with time and progressive recognition of the severity of the crisis, these partnerships became more focussed. More details on this aspect have been dealt with in subsequent sections where we explore quarterly publication trends in greater detail.

### 3.3. Authorship Trends and the Need for Gender Normalisation

**Figure 3:**
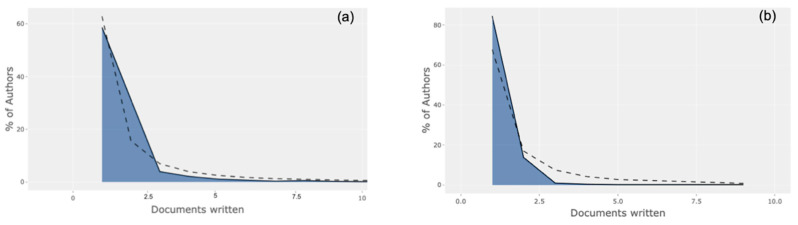
Analysis of authorship trends for (**a**) Group A COVID-19 and (**b**) Group C SARS 2003.

Figure 3 reveals authorship trends between COVID-19 (Group A) and SARS 2003 (Group C). More than half (58.3%) of all authors in 2020 contributed only one document to the collection, while 1% of all authors contributed five documents to the collection, a pattern quite close to the expected/theoretical Lotka curve (dotted line). However, in Group C, a more skewed curve was visible, revealing >80% of authors contributing only one document to the collection and 0.2% of authors contributing five documents.

An incredibly powerful measure of the pandemic’s impact on working women in science is lost in the collections, since article metadata do not capture gender metrics. We tried to manually scan all 75,608 author names in our collections, but naming conventions can rely heavily on demographics, history and geographical regions. We are now working towards building a strategy to identify gender from first names and contextual information. In all four quarters of 2020, about 80% of authors published single papers in the respective collections, as expected from the Lotka curves, and we decided to investigate the range of research thematics across the four quarters, as described in the next section. The collaboration index also dropped to its lowest during Q2, after which it rose steadily until Q4, providing evidence that the second quarter of the pandemic year witnessed a change in the collaborative tendencies of authors. This metric does not fully take into account the importance of the paper in its scientific community, but the relative contributions of its co-authors. This is important, since neglecting co-author information can inhibit the quantification of an individual researcher’s achievements and give others undue credit [22,23]. However, the rise in the number of keywords with a simultaneous decrease in the collaboration index prompts a rigorous assessment of individual keywords, as has been attempted in the next section, offering a more efficient bibliometric analysis.

### 3.4. Diversity of Research Thematics Reveals Scientific and Societal Priorities

**Figure 4:**
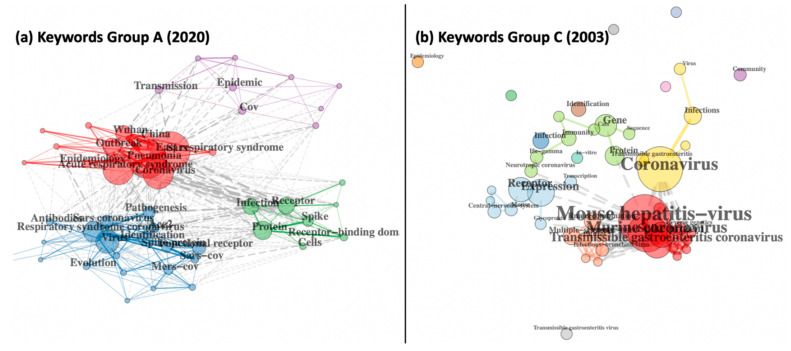
Keyword co-occurrence networks for (**a**) Group A COVID-19 and (**b**) Group C SARS 2003 collections.

Figure 4 depicts co-occurrence networks mapping the top keywords in Group A and Group C, and clear patterns emerge from clusters in both SARS and COVID-19 that reveal how distinctly the scientific community addressed the outbreaks, strongly governed by public sentiment and responses. Each network shows specific keywords as network nodes, while edges represent two keywords that occur together in the same document. Nodes are coloured by the clusters they belong to, and edges are coloured based on whether they connect nodes within or between clusters. If an edge connects nodes within a cluster, it is coloured the same as the corresponding cluster. Grey edges connect nodes between clusters, and these connections are distinctly more in Figure 4a representing Group A COVID-19. At the turn of the millennium, most SARS-related research involved viral infections in murine, equine, porcine or human models, and the use of gene/protein sequences was also emerging, but in complete isolation from other research clusters in the network (note the very few grey edges connecting clusters in Figure 4b, as well as the isolated clusters of Community and Epidemiology that are disconnected from the rest of SARS research). In contrast, research in the first 100 days of COVID-19 had already advanced into wider aspects such as management, mortality, epidemiology and human transmissions, for all kinds of respiratory syndromes focussing on diverse location-based geo-specific outbreaks. The strong overlap between all clusters reveals the huge connection between researchers and an interdisciplinary outlook towards the pandemic. In the first 100 days of 2020, the focus of research was maximally on the outbreak in Wuhan and its epidemiology (red), followed by disease pathogenesis (blue), and viral biology (green), while work on its transmission, mortality and management strategies was beginning to emerge (small purple cluster). Detailed investigation of the keyword data further reveals the extent to which IOT and big data contributed to each collection, with almost 150 keywords related to big data, internet, social media, rumour spreading, societal perceptions, artificial intelligence, machine learning, online education and whole genome comparisons in Group A, as compared to less than ten keywords in Group C, relating to the roles of computer communication networks, mobile phone antennas and image analysis, reflective of early 2003 when genomics, AI and ML were all yet to emerge and social media was simply non-existent. Changing social ideas and shifting societal priorities are further evident from an assessment of the 814 vs. 365 keywords of Group A and Group C, respectively. During the first 100 days of the COVID-19 pandemic, the published literature included keywords such as on social distancing, air travel restrictions, civil liberties, austerity and the allocation of scare resources, and “suspending classes without stopping learning”. In addition, this collection also has mental wellbeing-related keywords, such as fear psychosis, anxiety, attitudes, burnout, telehealth, telemedicine, women, workplaces, community outreach and self-management. In contrast, the scientific corpus in the initial days of the 2003 SARS epidemic only had three keywords related to public or social issues, namely public health, bioterrorism and social status, none of which suggest even a fraction of the extensive focus these issues have received and continue to receive in the ongoing global COVID-19 pandemic.

**Figure 5:**
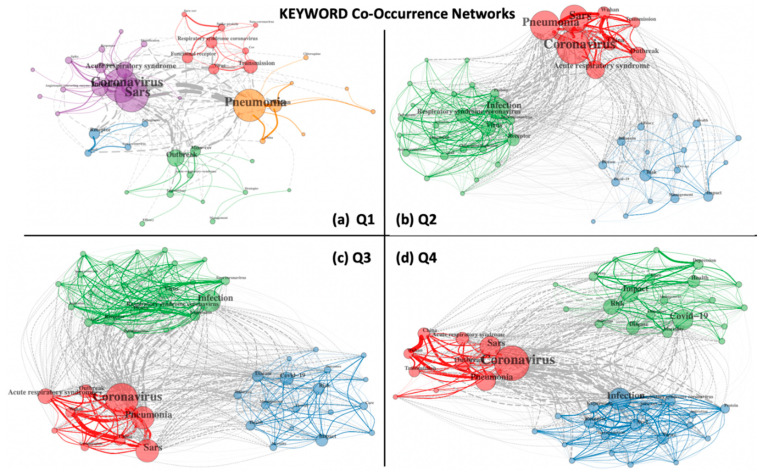
Keyword co-occurrence networks during the pandemic for (**a**) Q1, (**b**) Q2, (**c**) Q3, and (**d**) Q4.

This pattern of a distinct societal and social focus during COVID-19 prompted us to explore how the potential priorities (in terms of keywords in published corpus documents) have evolved over the entire year of 2020, and this was explored by comparing keyword co-occurrence maps for all four quarters as described in Materials and Methods. Figure 5 depicts these four maps and the first quarter of 2020 (Q1), and the focus of research was maximally on disease emergence, diagnosis and responses (purple), followed by treatment and side-effects (yellow), the outbreak of the pandemic (green), transmission (red) and pathogenesis (blue), and viral biology (green), while work on management strategies and potential drugs was beginning to emerge. By the second quarter (Q2), the global danger had been recognised and documents revealed three major clusters: grappling with global transmission and outbreak (red), pathogenesis, infections or treatment (green), and finally management and efficacy, suggesting that research into the societal impact of the pandemic (stress, risk, children, care and therapy) started to appear on the map (blue cluster), although kt was still not fully integrated with the other two tightly connected clusters (note the density of grey edges between clusters). During the third quarter of 2020 (Q3), this blue cluster integrated with the red and green clusters, revealing the increasing impact of societal concerns on pandemic-associated research. Keywords such as “depression” and “fear” emphasised the extent to which the public was impacted by the pandemic, while keywords such as AI, ML and twitter trends revealed the focus on “big data” driven machine learning initiatives. By the last quarter of 2020 (Q4), the three clusters became strongly integrated, but the secondary impact cluster had taken a more central position (green) on the map, with keywords such as “impact” becoming most prominent along with the emergence of aspects of mental health, wellbeing, anxiety, and performance. The term “vaccine” was observed in both the Group A and C collections, but later quarters of 2020 witnessed the emergence of a large number of vaccine-related terms and viral evolution (numbering 51, 80 and 111 from Q2 to Q4) encompassing vaccine design, development, trials, dose, rationing, targets, safety, uptake, efficacy, acceptance, adjuvants, subunits and types, among others. In an effort to investigate the impact of societal inequalities, we found >25 keywords related to poverty. This was further supported by the observation that publications from economically backward regions with relatively lesser known institutions and authors were mostly peripheral in our networks rather than central. As shown in the next section, economically advanced countries and rich/branded institutors took up significantly more central locations in several temporal networks as compared to low-income countries. 

### 3.5. Trends in Collaboration across Institutional and National Boundaries

**Figure 6:**
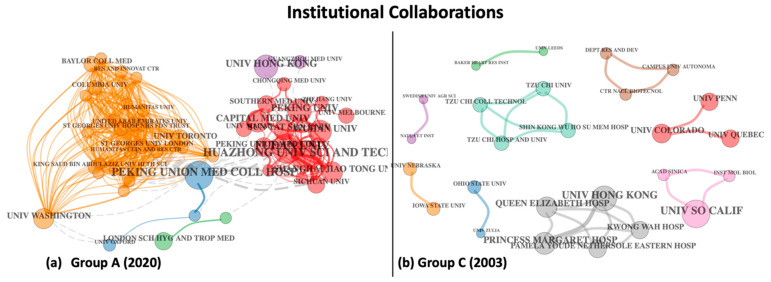
Institutional collaboration networks for (**a**) Group A COVID-19 and (**b**) Group C SARS 2003.

Figure 6 depicts the institutional collaboration networks for both COVID-19 and SARS groups, both clustered by a unified approach where a weighted variant of modularity-based community detection has been used to identify the institutions that have the maximum collaborating authors.

The institutional affiliation patterns in Figure 6 are dramatically different for Group A (COVID-19) as compared to Group C (SARS), with the former showing three fairly well-connected clusters in contrast to nine small clusters in the latter, which are entirely isolated from each other, suggesting very little collaboration between major research groups steering the investigations immediately following the 2003 outbreak as compared to the 2020 outbreak. The largest cluster in Group A (orange) represents primarily American organisations, followed by Chinese institutes (red), but this cluster also includes the University of Melbourne (collaborating with Peking University) and these two major clusters are indirectly connected via a third smaller cluster (in blue) representing the University of Oxford and Peking Union Medical College Hospital. Figure 6b depicts the SARS 2003 collections with very distinct regional clusters, with the largest cluster (in grey) representing a set of institutions based in Hong Kong. Not surprisingly, most of these clusters are either limited to the Far East or North America, as reflected by the geographical extent of the SARS-CoV epidemic in 2003.

Country collaboration networks for both groups showed very similar patterns, with Group A having authors from several countries led by a strong collaboration between the United States of America (USA) and China, but also having authors from Denmark, Pakistan, Ghana and Canada, among others. Even the smallest collaboration clusters in COVID-19 reflected diverse regional representation, e.g., Japan with Honduras, Nepal and Colombia. Meanwhile, the publications at the onset of SARS were from five economically powerful, developed countries of the world, which were, surprisingly, further divided into two isolated clusters (UK–Australia and USA–Taiwan–Canada). These patterns further reiterate the extent to which COVID-19 has bridged scientific inequality, enabling new, more resilient researcher networks worldwide. Greater access to data, the sharing of critical technology, and local insights have enabled researchers to better understand how the pandemic is impacting societies in different ways in different places. This, in turn, has allowed the scientific community during COVID-19 (unlike SARS) to evaluate and bring out the best possible interventions to address the problems at various levels and improve the resilience of society at large.

**Figure 7:**
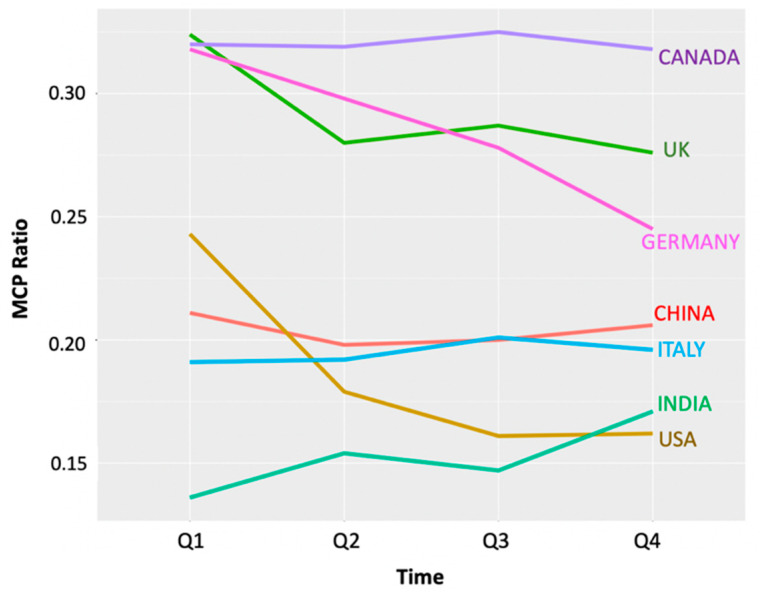
MCP Ratio for the six most collaborative countries during the year of the pandemic.

As explained in the Introduction, the MCP Ratio is a metric to quantify a country’s international collaboration. We used this metric in the four temporal quarterly country collaboration networks for COVID-19, as depicted in Figure 7. Only six countries were common across the top ten most productive countries in the four collections, and these are the only ones depicted in this line plot. The highest MCP Ratios were observed for Canada, the UK and Germany consistently through the year, while China, the USA, Italy and India were slightly lower in terms of MCP Ratios. India was observed to have the maximum rise in MCP Ratio over time, followed by China, while Canada maintained the highest value across the four collections. These trends emphasise the importance of data access and data sharing between nations. Germany showed a sustained decrease in the MCP Ratio after the first quarter, suggesting that it was at the top of the game when the pandemic broke but was soon brought to a near closure of all international collaborations, arising (presumably) from the severe societal disruption and nation-wide impact of the pandemic, with some of the world’s highest mortalities during the first and second quarters of 2020.

**Figure 8:**
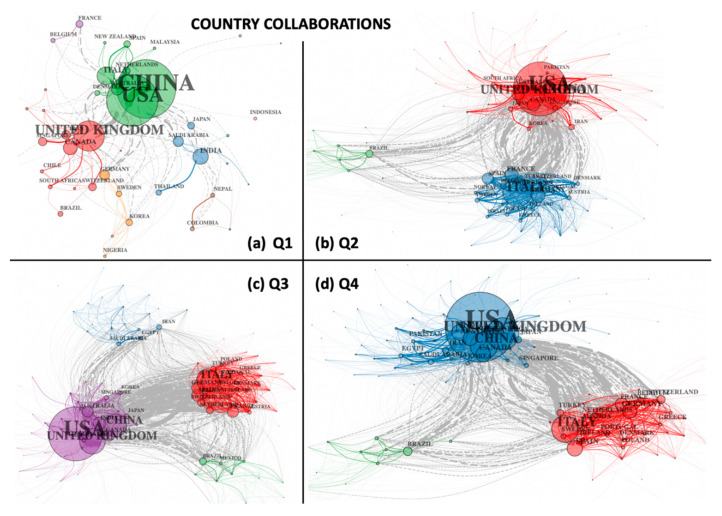
Quarterly country collaboration networks for 2020 (**a**) Q1, (**b**) Q2, (**c**) Q3, and (**d**) Q4.

More insights into these trends can be observed in Figure 8, which provides a detailed breakup of the country collaboration networks over the four temporal networks for 2020. Nodes in these networks represent countries, while edges represent co-authors of documents from both countries. The width of the edges represents the number of authors common to two connecting nodes, while colours (of nodes and edges) represent clustering patterns in the data. As with other network figures, grey edges represent connections between clusters. In the first quarter (Q1), China, the USA, and the UK dominated the map, while the second quarter (Q2) witnessed the union of these two clusters into one (red cluster), along with the emergence of a strongly interconnected second cluster of other European countries (blue), as well as a peripheral cluster (green) of Latin American nations. In the third quarter (Q3), the two main clusters became strongly interconnected (grey edges connecting red and purple clusters), while the Latin American cluster (green) remained intact, as a new cluster of Middle Eastern nations emerged (blue). Many new developing countries joined the largest (purple) cluster, while all four clusters developed new links and overlaps. In the last quarter (Q4), the Middle Eastern cluster merged with the largest cluster (now blue in colour), followed by the European (red) and Latin American (green) clusters, both of which remained intact, while links between countries become even more pronounced.

Institutes were ranked on the basis of the number of documents they contributed to the four quarterly collections, and the quarterly COVID-19 collaboration networks for institutes across the year 2020 showed similar patterns as were observed in Figure 8. Seven institutes appeared consistently in the list for each quarter, with the Huazhong University of Science and Technology at the top, followed by Wuhan University (located in the epicentre of the pandemic), at Rank 2 from Q1–Q3. In the last quarter (Q4), Wuhan was overtaken by the University of Toronto, which featured in the third place from Q1–Q3.

## 4. Discussion

A temporal bibliometric analysis of coronavirus-related research as presented in this work offers a near-real time glimpse into how the pandemic is impacting both science and societies across the globe. This work also brings out the benefits of extremely advanced technical capacity as well as the extraordinary amount of data available in 2020, and its impact on the policy process. The temporal bibliometric networks shown here identify several interesting trends in academic publishing during two major coronavirus epidemics of SARS-CoV (2003) and COVID-19 (2020).

The annual scientific production for coronavirus-related research peaked during the SARS and MERS epidemics, and then again during the current COVID-19 pandemic, but we noted clear distinctions between the COVID-19 and SARS-CoV outbreaks (first 100 days) in terms of a higher number of documents and authors, wider keyword co-occurrence, and greater collaboration patterns between countries and organisations during 2020 as compared to 2003 (Table 1; Figure 2, Figure 3, Figure 4 and Figure 6). As mentioned earlier, one reason for this could be the limited nature of the SARS-CoV epidemic, as reflected in the list of the most productive countries (Far East and North America) that were also the ones most afflicted by the outbreak at the time. However, equally, the diversity of thematics in 2020 and the consistently higher international integration observed immediately after the COVID-19 outbreak suggest that the global scientific community came together to collaborate in order to address the crisis. This was further supported by Lotka’s law curves that showed how authors researching COVID-19 in 2020 were more “dedicated” to publishing than researchers of SARS back in 2003, when more than 80% of authors contributed to only one publication in the collection. At the beginning of the year, most publications came from the epicentre of the pandemic—China. However, by the end of the year, although global collaborations increased, most of them were among researchers in the Western world. Research from China and the developing world became less significant as it became clearer that the pandemic was a global concern.

Keyword co-occurrence networks showed that initial COVID-19 research was far more interdisciplinary, and more importantly, the 2020 scientific corpus revealed the extent to which IOT and big data have contributed to the collection, with hundreds of keywords related to smart phones, web-cams, social media trend-monitoring platforms, artificial intelligence (AI), deep learning and genomics. These trends were further resolved in real-time by comparisons between keyword co-occurrence and thematic clusters across the four quarters of 2020. Documents in the second to fourth quarters of the 2020 corpus revealed dozens of keywords relating to the usage of smartphone apps and mobile-based interventions or technologies (such as smartphone microscopes, readers, mobile-based learning, screening tools, surveys, consultations and others). Over the year, we saw research interest diversifying from disease biology to its secondary impact on people’s mental health and wellbeing. By the end of the year, impact-related research had become common. Changing social perceptions and shifting societal priorities were further evident from an assessment of the 2020 scientific corpus, which showed an increasing number of keywords from Q1 to Q4 relating to psychological wellbeing, emotional health, social responsibility and societal participation in combating the outbreak at all levels, across all ages and strata of society. Interestingly, none of these keywords were observed in the SARS-CoV outbreak, when big data technologies such as genomics, AI and ML were all yet to emerge and social media was simply non-existent. In addition, the four quarters of 2020 also reflect an increasing number of keywords (numbering hundreds) related to societal management, e-learning, online communication, working from home, fear psychosis, anxiety, rehabilitation, burnout, women, workplaces, community outreach and self-management. 

Over the course of the pandemic year itself, we saw a steadily increasing interest in publishing COVID-19 research, with several journals documenting a boom in publications at the beginning of the year, so much so that they occupied a core position in the first quarter. However, after March 2020, most of those core quarter 1 journals disappeared from the collection altogether for the rest of the year. The *BMJ* produced a large number of documents throughout the year. However, its impact measured by the h-index remained consistently below that of other journals that were publishing about half the number of papers, such as the *Lancet* and *Journal of Medical Virology*. The much-discussed hydroxychloroquine paper was retracted by the *Lancet* in June 2020. The h-index for the *Lancet* fell rapidly after June 2020, reducing the h-index difference among these top journals. 

Green spaces took on new importance across the world at this time of crisis, especially in urban areas, as evident from an increasing number of keywords relating to green spaces and urban landscaping (from Q1:Q4 these were 9; 26; 70; 81 keywords), reinforcing the need for (and health benefits of) accessible public parks and forested areas. Such benefits of green spaces can be factored into post-COVID urban planning policies.

## 5. Conclusions

In this work, we compare the past and present coronavirus outbreaks in terms of published literature, and attempt to reflect upon the impact of the crises on science and societal priorities. Apart from taking a social perspective, we also touch upon limitations, as well as aspects of bibliometry that are generally not explored in typical studies in this area. For example, several previous works on bibliometric investigations into the pandemic have explored papers originating from specific countries or languages [24,25], or the pandemic from a subject specific perspective [26], or the exploration of the top N papers [27]. More general pandemic-related investigations have highlighted aspects such as the most productive authors, the most cited papers, the value of pre-prints, etc. [28,29,30], but we believe that a true bibliometric investigation should be able to explore the past in the context of the present, and to provide future perspectives that may be useful for historians or policy makers, especially when dealing with a crisis as global and catastrophic as COVID-19. The present work has enabled us to conceptually expand this field of work, overarching beyond earlier works of similar nature.

Another limitation of most bibliometry studies is the missing handles for societal inequalities that cannot be proxied measurably from scientific publications, but we tried to assess this by two separate methods. On one hand, we identified about 20–25 keywords and research thematics related to terms such as poverty, societal inequality, and the liability of poorness, while on the other hand, we attempted to measure publications from economically backward regions with relatively lesser known institutions and authors in our collections. We noted that documents from the highest-income areas (economically advanced countries) had significantly more central locations in several temporal networks as compared to low-income countries. However, these trends appeared to be diminishing by Q4 and the future may hold surprises that we are currently in the process of predicting. For instance, it has been noted that reduced economic activity and travel during the pandemic has reduced air pollution and deaths from traffic accidents and crashes, and this may be indirectly inferred from the scores of keyword terms in the 2020 collections that were found to be related to travel restrictions (for, e.g., travel bans, travel history and behaviour), but the published corpus does not yet allow us to quantify this aspect fully. We are currently exploring in more detail the >25,000 keywords that we have collated over the course of this work.

A critical feature missing from the current analysis is the gender ratios, as emphasised in the text already. It has been predicted that it may take about two decades before the number of women on scientific papers is equal to the number of men. We undertook a manual inspection for trends between 2003 SARS and the current 2020 pandemic, and found strong skews that mask huge amounts of variation and merit a more dedicated analysis of the collections, currently underway in our laboratory. We are trying to identify the numbers of women authors in each collection, their rates of publishing, the extent to which women are outnumbered by men across subject areas, and more.

In summary, the trends observed in this work provide valuable insights into how academia responds to a global calamity, and how societal impact and public responses steer research worldwide. We may learn useful lessons on the real-world importance of ensuring diversity, accessibility, and quality in scientific thought. The analysis of quarterly temporal networks during the pandemic also emphasised the necessity and need to include the time dimension in such investigations, and how often we miss out on perspectives that enable us to be better prepared for recurrent stressors. We also reiterate that it is our collective responsibility to use the pandemic associated “big data” and the exponentially increasing new wealth of information for a better world.

## Figures and Tables

**Table 1:** Comparison of Data Collection Groups A, B and C.

Description	Group A	Group B	Group C
**PERIOD**	2020	2001–2020	2003
**DOCUMENTS:**			
Sources (Journals, Books, etc.)	291	3598	101
Documents	1017	23,818	186
References	7624	342,431	4086
Keywords	814	23,867	365
Types of Documents	12	24	9
**AUTHORS:**			
Authors	2764	49,216	510
Authors of single-authored documents	158	1496	41
Authors of multi-authored documents	2606	47,720	469
**AUTHOR COLLABORATIONS:**			
Single-authored documents	294	2458	59
Documents per Author	0.368	0.484	0.365
Authors per Document	2.72	2.07	2.74
Co-Authors per Document	4.73	5.75	3.27
Collaboration Index	3.6	2.23	3.69

## Data Availability

The data presented in this study are available on request from the corresponding author.
